# Plant growth regulator diethyl aminoethyl hexanoate suppresses *Aspergillus flavus* growth and aflatoxin biosynthesis through disruption of redox homeostasis

**DOI:** 10.1128/aem.00970-26

**Published:** 2026-09-14

**Authors:** Kai Ouyang, Lei He, Yarong Zhao, Siyi Tan, Guanxian Chen, Yingwen Wu, Xinyu Han, Xiaorong Wan, Qingqing Zhi

**Affiliations:** 1 College of Agriculture and Biology, Guangzhou Key Laboratory for Research and Development of Crop Germplasm Resources, Zhongkai University of Agriculture and Engineering 47894, Guangzhou, China; 2 Key Laboratory of Green Prevention and Control on Fruits and Vegetables in South China, Ministry of Agriculture and Rural Affairs, Zhongkai University of Agriculture and Engineering 47894, Guangzhou, China; 3 School of Food Science, Guangdong Polytechnic of Science and Trade 603915 , Guangzhou, China; 4 Institute of Quality Standard and Monitoring Technology for Agro-Products, Guangdong Academy of Agricultural Sciences 117866, Guangzhou, China; Michigan State University, East Lansing, Michigan, USA

**Keywords:** aflatoxin, *Aspergillus flavus*, diethyl aminoethyl hexanoate, plant growth regulator, antifungal activity, reactive oxygen species

## Abstract

Aflatoxin B₁ (AFB₁), produced by *Aspergillus flavus*, is a major threat to food safety and human health. In this study, we report that diethyl aminoethyl hexanoate (DA-6), a plant growth regulator, exhibits strong antifungal and anti-aflatoxigenic activities against *A. flavus*. DA-6 significantly inhibited mycelial growth, conidial germination, and sclerotia formation in a dose-dependent manner, while also effectively suppressing fungal colonization in stored rice bran and maize. Microscopic and biochemical analyses revealed that DA-6 disrupted plasma membrane integrity, leading to cytoplasmic leakage and hyphal collapse. Further analyses demonstrated that DA-6 caused severe oxidative-redox imbalance, characterized by excessive reactive oxygen species (ROS) accumulation and impaired antioxidant defenses, which were accompanied by mitochondrial dysfunction, reduced membrane potential, and ATP depletion. Transcriptomic profiling confirmed a global repression of the aflatoxin biosynthetic gene cluster (including *aflR* and *aflS*) and key developmental regulators (*laeA* and *veA*), resulting in the near-complete cessation of AFB₁ production. Additionally, functional characterization of two highly responsive Zn₂Cys₆ transcription factors (AFLA_059960 and AFLA_084720) further supported the involvement of oxidative stress adaptation pathways in the fungal response to DA-6-induced stress. Collectively, these findings provide mechanistic insights into the antifungal action of DA-6 and highlight its potential application for controlling fungal contamination and aflatoxin risks in agricultural commodities.

**IMPORTANCE:**

Plant growth regulators (PGRs) are widely used in agriculture, yet their potential effects on fungal physiology and mycotoxin biosynthesis remain largely unexplored. In this study, we demonstrate that diethyl aminoethyl hexanoate (DA-6) caused extensive transcriptional repression of fungal developmental regulators and the aflatoxin biosynthetic gene cluster, accompanied by severe oxidative-redox imbalance and mitochondrial dysfunction. These findings reveal a previously unrecognized antifungal application of PGRs and provide new insights into their potential use for controlling toxigenic fungi and mycotoxin contamination in food and agricultural systems.

## INTRODUCTION

*Aspergillus flavus* is a ubiquitous environmental fungal saprophyte that readily contaminates crops and agricultural commodities, including maize, peanuts, and grains during preharvest, storage, and processing stages (1, 2). *A. flavus* is notorious for producing hazardous aflatoxins (AFs), among which AFB_1_ poses the greatest health risk due to its pronounced hepatotoxic, teratogenic, and carcinogenic properties (3, 4). Contamination of crops and their derived products by *A. flavus* and AFs not only causes severe spoilage and quality deterioration but also poses major risks to food safety and public health, resulting in substantial economic losses worldwide (1, 5). Given the difficulty of eradicating *A. flavus* from the environment and the persistence of its reproductive structures and secondary metabolites, the development of efficient antifungal agents and control strategies remains an urgent global priority.

Currently, chemical fungicides remain the most common strategy for controlling *A. flavus* and aflatoxin contamination due to their cost-effectiveness and high efficiency. However, their large-scale application is restricted by concerns regarding chemical residues in food chains, environmental pollution, and the emergence of fungicide resistance (6, 7). While natural plant extracts like essential oils and phenolics have been explored, their industrial application is often hampered by high costs or instability of these formulations. In this context, certain agricultural chemicals with low toxicity and high biodegradability present a promising alternative. Plant growth regulators (PGRs) are a class of bioactive small molecules widely used in agriculture to regulate plant growth, development, and responses to abiotic and biotic stresses (8, 9). Beyond their canonical roles in plants, accumulating evidence indicates that some PGRs can directly influence phytopathogenic fungi, influencing fungal physiology, highlighting their potential as multifunctional agricultural compounds for disease management (10–12). For example, methyl jasmonate (MeJA) inhibits *A. flavus* growth and aflatoxin biosynthesis, whereas indole-3-acetic acid suppresses *Magnaporthe oryzae* growth and spore germination (13, 14). Diethyl aminoethyl hexanoate (DA-6) is a widely used PGR in agriculture that promotes plant growth and has recently been reported to enhance plant tolerance to abiotic stresses (15–17). Given its established agricultural applications, favorable safety profile, and potential multifunctionality, DA-6 represents an attractive candidate for repurposing as an antifungal compound. However, whether DA-6 directly affects phytopathogenic or food-associated fungi and their secondary metabolism remains largely unknown.

In this study, we investigated the activity of DA-6 against growth of *A. flavus* and its ability to suppress aflatoxin biosynthesis. Through integrated physiological, biochemical, and transcriptomic analyses, we found that DA-6 induced ROS accumulation while repressing antioxidant defenses, which subsequently caused mitochondrial dysfunction, energy depletion, and impairment of fungal development and toxin biosynthesis. This work expands the biological understanding of DA-6 from a plant growth regulator to a potential antifungal compound and provides a new perspective for developing multifunctional agricultural inputs to control fungal contamination and aflatoxin accumulation.

## MATERIALS AND METHODS

### Chemicals, strains, and culture conditions

Eight commonly used plant growth regulators including research-grade diethyl aminoethyl hexanoate (CAS: 10369-83-2, 98% purity), brassinolide (CAS: 72962-43-7), salicylic acid (CAS: 69-72-7), abscisic acid (CAS: 21293-29-8), indole-3-acetic acid (CAS: 87-51-4), 2-nitrophenol sodium salt (CAS: 824-39-5), 2-naphthylacetic acid (CAS: 581-96-4), and ethephon (CAS: 16672-87-0) were purchased from Shanghai Aladdin Reagent Co., Ltd. (Shanghai, China).

The *A. flavus* strain NRRL3357, a well-characterized aflatoxin-producing reference strain, was originally obtained from the laboratory of Prof. Gary Payne (North Carolina State University) and has been maintained in our laboratory as glycerol stocks. Strain was cultivated on potato dextrose agar (PDA, Difco) at 30°C for 5 days. The conidia were harvested by washing with 0.05% (vol/vol) Triton X-100 and resuspended in the same solution. The suspension was adjusted to 1 × 10^8^ conidia/mL with a hemocytometer.

### Antifungal activity assay of plant growth regulators

The antifungal activities of eight plant growth regulators against *A. flavus* were preliminarily evaluated using a broth microdilution-based assay to determine fungal growth inhibition in 24-well plates. Stock solutions of the plant growth regulators (except DA-6) were prepared according to their solubility in sterile distilled water or methanol, and corresponding solvent controls were included to rule out solvent effects. DA-6 (98% active ingredient; liquid formulation) was directly added to the culture medium without additional organic solvents. The *A. flavus* conidial suspension in potato dextrose broth (PDB, Difco) was adjusted to 1 × 10⁶ conidia/mL and dispensed into 24-well plates (995 μL per well). Subsequently, 5 μL of the respective stock solutions of the tested plant growth regulators (except DA-6) was added to each well to achieve the desired final concentrations, resulting in a final volume of 1 mL per well and a final conidial concentration of approximately 9.95 × 10⁵ conidia/mL. After incubation at 30°C for 48 h in PDB under static conditions, fungal growth in each well was visually inspected.

The MIC of DA-6 was determined using a broth dilution method with slight modifications. Briefly, the *A. flavus* conidial suspension was prepared in PDB and adjusted to approximately 1 × 10⁶ conidia/mL. The suspension was dispensed into 24-well plates, and liquid DA-6 was directly added to achieve final concentrations of 0, 0.1%, 0.2%, 0.4%, and 0.8% (vol/vol), corresponding to approximately 889, 1,778, 3,555, and 7,111 mg/L active ingredient, in a final volume of 1 mL per well. The final conidial concentration was approximately maintained at 1 × 10⁶ conidia/mL. After incubation at 30°C for 48 h under static conditions, fungal growth was visually evaluated. The MIC was defined as the lowest concentration of DA-6 that completely inhibited visible growth.

### Measurement of fungal growth and development

For colony growth assays, 1 µL of conidial suspension containing approximately 10^3^ conidia was inoculated on PDA supplemented with DA-6 at final concentrations of 0%, 0.1%, 0.2%, 0.4%, 0.8%, and 1.6% (vol/vol). DA-6 was added to autoclaved PDA medium cooled to approximately 60°C, thoroughly mixed, and poured into Petri dishes before inoculation. Colony diameters were measured after incubation at 30°C for 7 days. Mycelial dry weight was determined by culturing *A. flavus* in 30 mL of PDB supplemented with the indicated concentrations of DA-6 and inoculated with conidia at a final concentration of 1 × 10⁶ conidia/mL. Cultures were incubated at 30°C and 200 rpm under standard laboratory conditions. After 6 days of cultivation, mycelia were harvested by filtration and weighed after drying at 80°C for 6 h.

The inhibition of conidial germination was measured as in a previous study with minor modifications (18). *A. flavus* conidia (final concentration: 1 × 10⁶ conidia/mL) were inoculated into 5 mL of PDB containing DA-6 at final concentrations of 0%, 0.1%, 0.2%, 0.4%, and 0.8% (vol/vol). Sterile coverslips were added for microscopic observation, and cultures were incubated at 30°C under static conditions. After 12 h, conidial germination was observed with a microscope (Olympus, Tokyo, Japan). Conidia were considered germinated when the germ tube length was equal to or exceeded the diameter of the conidium. The germination rate (%) = (germinated conidia/total conidia) × 100%.

Sclerotia production was measured as previously described with minor modifications (19). Briefly, *A. flavus* conidia were inoculated onto glucose minimum medium (GMM) agar supplemented with 2% (wt/vol) sorbitol. The GMM contained 6.0 g NaNO_3_, 0.52 g KCl, 1.52 g KH_2_PO_4_, 0.52 g MgSO_4_, 10 g glucose, 1 mL trace element solution, and 15 g agar per liter. The medium pH was adjusted to 6.5 prior to autoclaving. DA-6 was added to the medium after cooling to approximately 60°C prior to plate pouring. The cultures were incubated at 30°C for 7 days in the dark under static conditions. The surface mycelia and conidia were gently removed with 75% ethanol to facilitate visualization of sclerotia. Sclerotia production was evaluated by quantifying the total number of sclerotia per plate using ImageJ software.

To evaluate the oxidative stress tolerance of WT and transcription factor mutant strains, H_2_O_2_ and tert-butyl hydroperoxide (t-BOOH) were added to autoclaved PDA medium after cooling to approximately 50°C to obtain final concentrations of 10 mM and 2 mM, respectively. The supplemented PDA medium was then poured into sterile Petri dishes and allowed to solidify at room temperature before inoculation. A 1 µL aliquot of conidial suspension containing approximately 1 × 10^3^ conidia was inoculated onto the center of each plate. The plates were incubated at 30°C in the dark for 3 days, and fungal growth was evaluated by measuring colony diameter. Relative colony growth under stress conditions was calculated as the ratio of colony diameter under stress conditions to that under control conditions.

### Examination of aflatoxin production

Aflatoxin B_1_ (AFB_1_) production was measured using modified thin layer chromatography (TLC) analysis as previously described (20). *A. flavus* conidia were inoculated into 30 mL PDB at a final concentration of approximately 1 × 10⁶ conidia/mL, supplemented with DA-6 at final concentrations of 0%, 0.1%, 0.2%, 0.4%, and 0.8% (vol/vol). The cultures were incubated at 30°C with shaking at 200 rpm, and the mycelia and cultures supernatants were collected at different time points for AFB_1_ extraction. Equal amounts of mycelial biomass (0.1 g fresh weight) were used for intracellular AFB₁ extraction to ensure comparability among treatments. AFB₁ was extracted using chloroform as previously described, and the organic phase was evaporated to dryness prior to resuspension in acetone. The extract residue was then spotted onto Si250 silica gel plates. Separation was performed using chloroform-acetone (9:1, vol/vol) as the mobile phase, and the plates were examined under UV light at 254 nm. Standard AFB_1_ was obtained from Sigma.

### Rice bran and maize inoculation assays with DA-6

Fifteen grams of rice bran were placed in 250-mL Erlenmeyer flasks and sterilized at 121°C for 15 min. After cooling to room temperature, treatment suspensions (10 mL per flask) containing *A. flavus* conidia (1 × 10^5^ conidia/mL) and DA-6 were prepared. DA-6 was added at final doses of 20, 40, 60, and 80 µL/g rice bran, corresponding to approximately 17.8, 35.6, 53.3, and 71.1 mg/g active ingredient rice bran, respectively. Sterile water was used as the control treatment. The flasks were gently mixed to ensure uniform distribution of conidia and DA-6 throughout the substrate. The treated rice bran samples were stored at room temperature and fungal colonization and sporulation were evaluated after 1 month and 6 months.

For the maize kernel assay, kernels were surface-sterilized by immersion in 0.05% sodium hypochlorite for 3 min, followed by soaking in 75% ethanol for 30 s, and finally rinsed three times with sterile distilled water. Twenty grams of sterilized maize kernels was transferred into sterile Erlenmeyer flasks. Treatment suspensions (3 mL per flask) containing *A. flavus* conidia (1 × 10⁵ conidia/mL) and DA-6 were prepared. DA-6 was added at final doses of 2.5, 5, 15, and 30 µL/g maize, corresponding to approximately 2.2, 4.4, 13.3, and 26.7 mg/g active ingredient, respectively. Sterile water was used as the control treatment. The flasks were gently shaken at 30°C for 30 min to ensure uniform distribution of conidia and DA-6 on the kernel surfaces. The treated maize samples were stored at room temperature, and fungal colonization and sporulation were assessed after 5 days and 6 months.

For both rice bran and maize kernel assays, conidia were recovered from 5 g samples by vigorous vortexing in 0.05% (vol/vol) Triton X-100 and counted using a hemocytometer.

### Scanning electron microscopy and transmission electron microscopy assays

Scanning electron microscopy (SEM) and transmission electron microscopy (TEM) analyses were conducted according to the descriptions of Liu et al. (21) and Li et al. (13) with slight modifications. *A. flavus* conidia were inoculated into 30 mL PDB and incubated at 30°C, 200 rpm for 12 h. DA-6 was then added to the liquid cultures to achieve final concentrations of 0.4% (1/2× MIC), 0.8% (MIC), and 1.6% (2× MIC) (vol/vol), respectively. The mycelia treated without DA-6 were used as the control group. Cultures were further incubated for 36 h, and mycelia were harvested and rinsed with 0.1 M phosphate-buffered saline (PBS, pH 7) for three times. The mycelia were fixed with 2.5% (vol/vol) glutaraldehyde solution and stored at 4°C overnight. Samples were washed three times with PBS.

For SEM analysis, the harvested mycelia were dehydrated through a graded ethanol series (30%, 50%, 70%, 80%, 90%, and 100%). Then, samples were freeze-dried, sprayed with gold, observed, and photographed under the Hitachi Regulus 8100 scanning electron microscope (Hitachi, Tokyo, Japan).

TEM samples were post-fixed with 2% osmium tetroxide, dehydrated through a graded ethanol series, embedded in epoxy resin, and sectioned into ultrathin slices. The sections were stained with uranyl acetate and lead citrate and observed using a transmission electron microscope (HT7800, Hitachi, Tokyo, Japan) at 80 kV.

### Measurement of cell membrane integrity and leakage

*A. flavus* conidia were inoculated into 30 mL PDB to a final concentration of 1 × 10^6^ conidia/mL supplemented with different concentrations of DA-6. The cultures were incubated at 30°C with shaking at 200 rpm for 24 h. The culture broth was centrifuged at 4,000 × g for 5 min to recover the supernatant. Cell membrane leakage was determined by measuring the electrical conductivity and the absorbance of the culture supernatant at 260 nm (*A*_260nm_) and 280 nm (*A*_280nm_), which reflect the leakage of intracellular nucleic acids and proteins, respectively, as previously described (22). For propidium iodide (PI) stain assay, mycelia were collected and stained with 10 μg/mL PI for 30 min at 37°C to assess cell membrane integrity. The mycelia were then observed under a fluorescence microscope (Eclipse Ni-E, Nikon, Japan), where red fluorescence resulting from PI staining was used as an indicator of compromised cell membrane integrity (23, 24). To assess ergosterol content, *A. flavus* conidia were inoculated into 30 mL PDB containing DA-6 at final concentrations of 0%, 0.4%, 0.8%, and 1.6% (vol/vol) and incubated at 30°C with shaking at 200 rpm for 48 h. Ergosterol content, expressed as a percentage of mycelial biomass, was determined as previously described (25).

### Analysis of DNA damage and autophagy

Nuclear morphological changes after DA-6 treatment were examined by 4,6-diamidino-2-phenylindole (DAPI, Beyotime Biotechnology, Shanghai, China) staining. *A. flavus* conidia were inoculated into PDB supplemented with 0.4% DA-6 and cultured for 48 h at 30°C. The mycelia were permeabilized and fixed with an appropriate fixing solution for 15 min at room temperature (26). Then, the mycelia were washed twice in distilled water and stained in the dark with 100 ng/mL DAPI for 15 min (DAPI, Sigma). For autophagy analysis, the mycelia were stained with the monodansylcadaverine (MDC, Beyotime Biotechnology, Shanghai, China) dye at 37°C for 30 min. Samples were then washed twice in PBS and viewed using a Nikon fluorescence microscope.

### Determination of reactive oxygen species accumulation and catalase, and superoxide dismutase activities

Reactive oxygen species (ROS) accumulation, together with the activities of antioxidant enzymes superoxide dismutase (SOD) and catalase (CAT), was used to assess oxidative stress levels and antioxidant defense capacity of *A. flavus* in response to DA-6 treatment. ROS accumulation in *A. flavus* mycelia was assessed using the fluorescent probe 2,7-dichlorodihydrofluorescein diacetate (DCFH-DA, Solarbio, Beijing, China) according to the manufacturer’s instructions. *A. flavus* conidia were inoculated into PDB supplemented with DA-6 at final concentrations of 0%, 0.2%, 0.4%, and 0.6% (vol/vol) and incubated at 30°C for 48 h. The collected mycelia were stained at 37°C for 30 min in the dark and washed twice with PBS. The ROS contents in *A. flavus* were measured with a fluorescence microscope and a microplate reader (SpectraMax Mini, Molecular Devices, USA). SOD and CAT activities were measured using commercial assay kits (Solarbio, Beijing, China) according to the manufacturer’s instructions and expressed as units per gram of mycelial dry weight (U/g FW). Briefly, mycelia collected after DA-6 treatment were homogenized and extracted using the reagents provided in the kits, and the absorbance values were determined using a microplate reader (SpectraMax Mini, Molecular Devices, USA).

### Determination of mitochondrial membrane potential and intracellular ATP content

Mitochondrial membrane potential (MMP) and intracellular ATP content were determined to evaluate mitochondrial function in *A. flavus* under DA-6 treatment. For MMP analysis, *A. flavus* conidia were inoculated into 30 mL PDB at a final concentration of 1 × 10⁶ conidia/mL and incubated with 0.4% (vol/vol) DA-6 at 30°C for 48 h. Cultures without DA-6 served as the control. Changes of MMP in *A. flavus* hyphae were detected by fluorescent dye JC-1 (Elabscience, Wuhan, China) according to the manufacturer’s instructions. The collected hyphae were stained with JC-1 at 37°C for 20 min, rinsed with PBS, and visualized under a fluorescence microscope. The hyphae treated with 10 µM carbonyl cyanide 3-chlorophenylhydrazone (CCCP) were used as a positive control.

For intracellular ATP determination, *A. flavus* conidia were inoculated into 30 mL PDB at a final concentration of 1 × 10⁶ conidia/mL supplemented with DA-6 at final concentrations of 0%, 0.2%, 0.4%, and 0.6% (vol/vol), followed by incubation at 30°C for 48 h. Intracellular ATP content was determined using an ATP assay kit following the manufacturer’s instructions. The assay is based on the luciferase luciferin bioluminescence reaction, in which the emitted luminescence intensity is proportional to the ATP concentration. Luminescence was measured using a microplate reader.

### RNA sequencing and data analysis

*A. flavus* conidia were inoculated into PDB supplemented with DA-6 at final concentrations of 0%, 0.1%, and 0.2% (vol/vol) and incubated at 30°C for 24 h. Three independent biological replicates were prepared for each treatment group. Mycelia were harvested immediately for RNA extraction using Trizol Reagent (Vazyme, Nanjing, China). RNA integrity was assessed using an Agilent 2100 bioanalyzer system (Agilent Technologies, CA, USA). High-quality RNA was used to prepare sequencing libraries with the Illumina TruSeq kit and sequenced on an Illumina Hiseq 2500 (Biomarker, Beijing, China). Raw sequencing reads were quality-checked, filtered, and mapped to the *A. flavus* NRRL3357 genome (GCF_000006275.2) using HISAT2 software (27). Gene expression level was normalized by calculating the number of Fragments Per Kilobase per Million reads mapped (FPKM) (28). Differential expression analysis was performed using the DESeq2 software (29). Genes with |log_2_ (fold change)| ≥ 1 and the false discovery rate (FDR) less than 0.05 were defined as significantly differentially expressed. The gene function annotation was conducted based on the GO and KEGG databases.

### Mutant construction

TF deletion mutants of AFLA_059960 and AFLA_084720, selected based on transcriptomic analysis, were generated as previously described (19). Briefly, approximately 1–1.5 kb upstream and downstream flanking regions of each target gene were amplified by PCR and fused with the *A. flavus argB* selectable marker. The resulting deletion cassettes were introduced into the TJES20.1 (Δ*ku70*, Δ*argB*) parental strain protoplasts. *A. flavus* protoplast preparation and the polyethylene glycol method were carried out according to a previously described (30). The schematic diagram of the homologous recombination strategy is shown in Fig. S3A. All primers for mutant construction were listed in Table S1. Diagnostic PCR, RT-PCR, and qPCR were conducted to identify putative transformants (Fig. S3B through D).

### Quantitative reverse transcription PCR

*A. flavus* conidia were inoculated into PDB supplemented with DA-6 at final concentrations of 0%, 0.1%, and 0.2% (vol/vol) and incubated at 30°C for 24 h. Total RNA was extracted from the harvested mycelia and cDNA was generated using the HiScript α Q RT SuperMix cDNA Synthesis kit (Vazyme, Nanjing, China). Ten DEGs showing different expression patterns were selected from the RNA-seq data for RT-qPCR validation, and the expression levels of AF cluster genes were also analyzed by RT-qPCR. Primer sequences for all genes are provided in Table S2. RT-qPCR assay was performed with the ChamQ SYBR Master Mix (Vazyme, Nanjing, China) with a CFX96 Touch Real-Time PCR Detection System (Bio-Rad, USA) according to the manufacturer’s instructions. Gene expression levels were normalized to *A. flavus β*-actin using the 2^−ΔΔCt^ method. Three independent biological replicates were analyzed for each treatment, and each biological replicate was measured using three technical replicates.

### Statistical analysis

All experiments were independently repeated at least three times, with each independent experiment including three parallel replicates for each treatment. Data were presented as the mean ± SD. Differences among groups were tested by one-way ANOVA followed by Dunnett’s multiple comparison test. Statistical significance was set at *P* < 0.05. GraphPad Prism 8.4.3 was adopted for statistical analysis and statistical chart production (GraphPad Software, USA).

## RESULTS

### DA-6 inhibits *A. flavus* growth and development through transcriptional repression of developmental regulators

**Fig 1:**
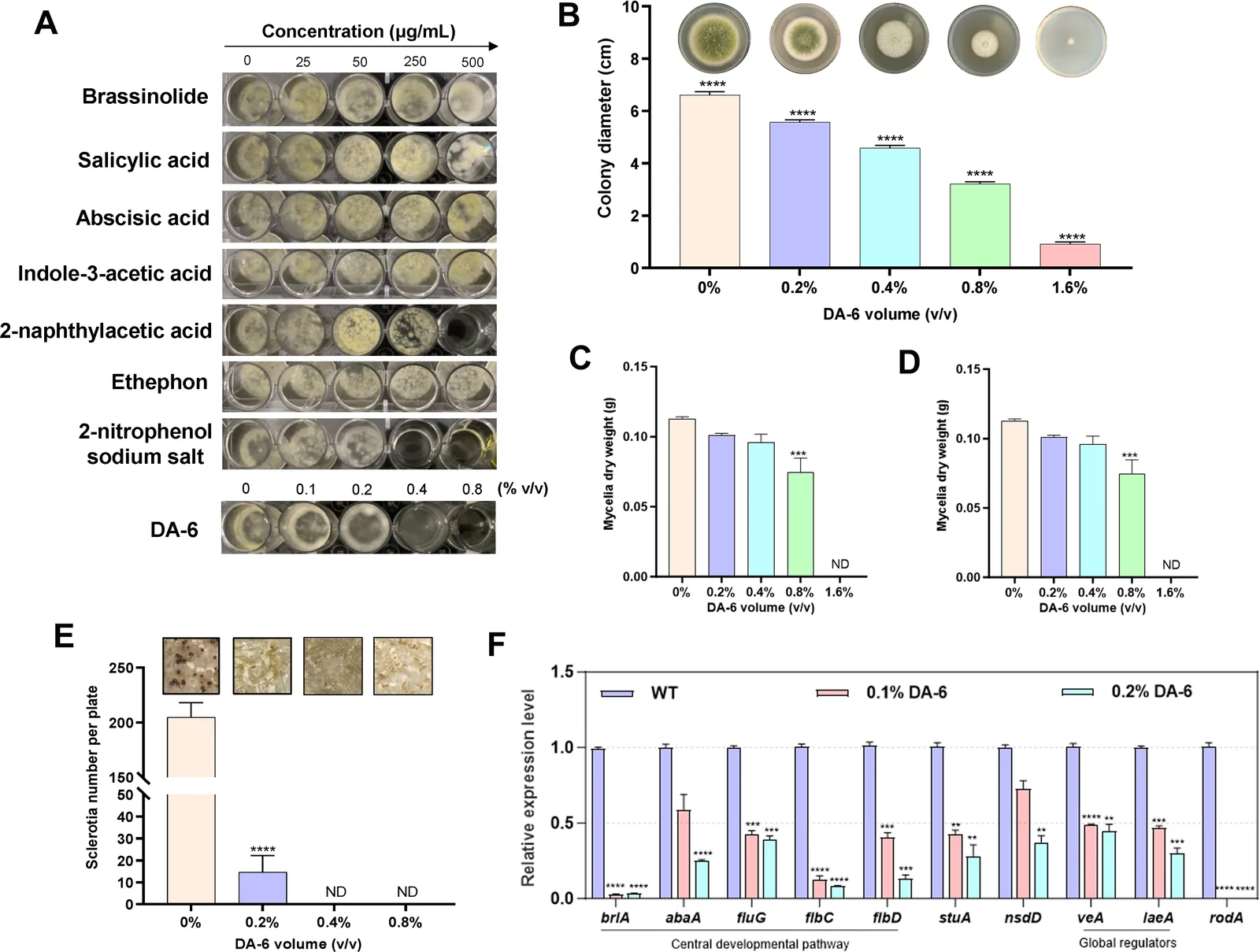
DA-6 inhibits the growth and development of *A. flavus*. (**A**) Preliminary screening of representative plant growth regulators against *A. flavus* under the tested conditions. (**B**) Effects of different concentrations of DA-6 on colony morphology and colony diameter of *A. flavus* grown on PDA plates for 7 days. (**C**) Effects of DA-6 on mycelial biomass in PDB medium. (**D**) Effects of DA-6 on conidial germination. (**E**) Effects of DA-6 on sclerotia formation and sclerotial production. (**F**) Transcriptomic changes of development-associated genes significantly regulated by DA-6 treatment (*P* < 0.05, |log_2_ FC| ≥ 1). Data are shown as mean ± SD, *n* ≥ 3. Statistical significance was determined by Student’s *t*-test compared with the control, ***P < 0.001, ****P < 0.0001; ND: not detected.

To identify plant growth regulators with antifungal activity against *A. flavus*, eight compounds were initially screened (Fig. 1A). Among them, DA-6 exhibited pronounced inhibitory activity against *A. flavus*, with a MIC of 0.8% (vol/vol) (equivalent to approximately 7,111 mg/L active ingredient). Considering its established agricultural applications and potential multifunctional roles, DA-6 was selected for further investigation. DA-6 treatment markedly inhibited colony growth on PDA plates in a dose-dependent manner, as evidenced by reduced colony diameter (Fig. 1B). Consistently, fungal biomass in PDB medium was significantly decreased following DA-6 treatment (Fig. 1C). DA-6 also strongly suppressed fungal developmental processes. Conidial germination was significantly inhibited at 0.2% DA-6 (approximately 1,778 mg/L active ingredient) and almost completely blocked at higher concentrations (Fig. 1D). Similarly, sclerotia formation was dramatically reduced by 0.2% DA-6 and completely abolished at concentrations ≥0.4% (vol/vol) (Fig. 1E).

To gain insights into the inhibitory mechanisms, transcriptomic analyses were conducted under 0.1% and 0.2% DA-6 treatment, revealing 3,133 and 4,502 DEGs, respectively, compared with the control (Fig. S1A). qRT-PCR validation of representative genes showed expression patterns consistent with the transcriptomic data (Fig. S1B). Notably, multiple genes involved in fungal development and conidiation were significantly downregulated by DA-6 treatment (Fig. 1F), including genes associated with the central regulatory pathway of conidiation (*brlA* and *abaA*), upstream activation of asexual development (*fluG*, *flbC,* and *flbD*), the conidial hydrophobin gene *rodA*, and the developmental regulators (*stuA* and *nsdD*). In addition, *veA* and *laeA*, two key regulators that coordinate fungal development and secondary metabolism (31), were also markedly repressed. These findings indicate that DA-6 suppresses fungal growth and developmental differentiation at least partially through transcriptional repression of developmental regulatory pathways.

### DA-6 suppresses *A. flavus* infection in rice bran and maize

**Fig 2:**
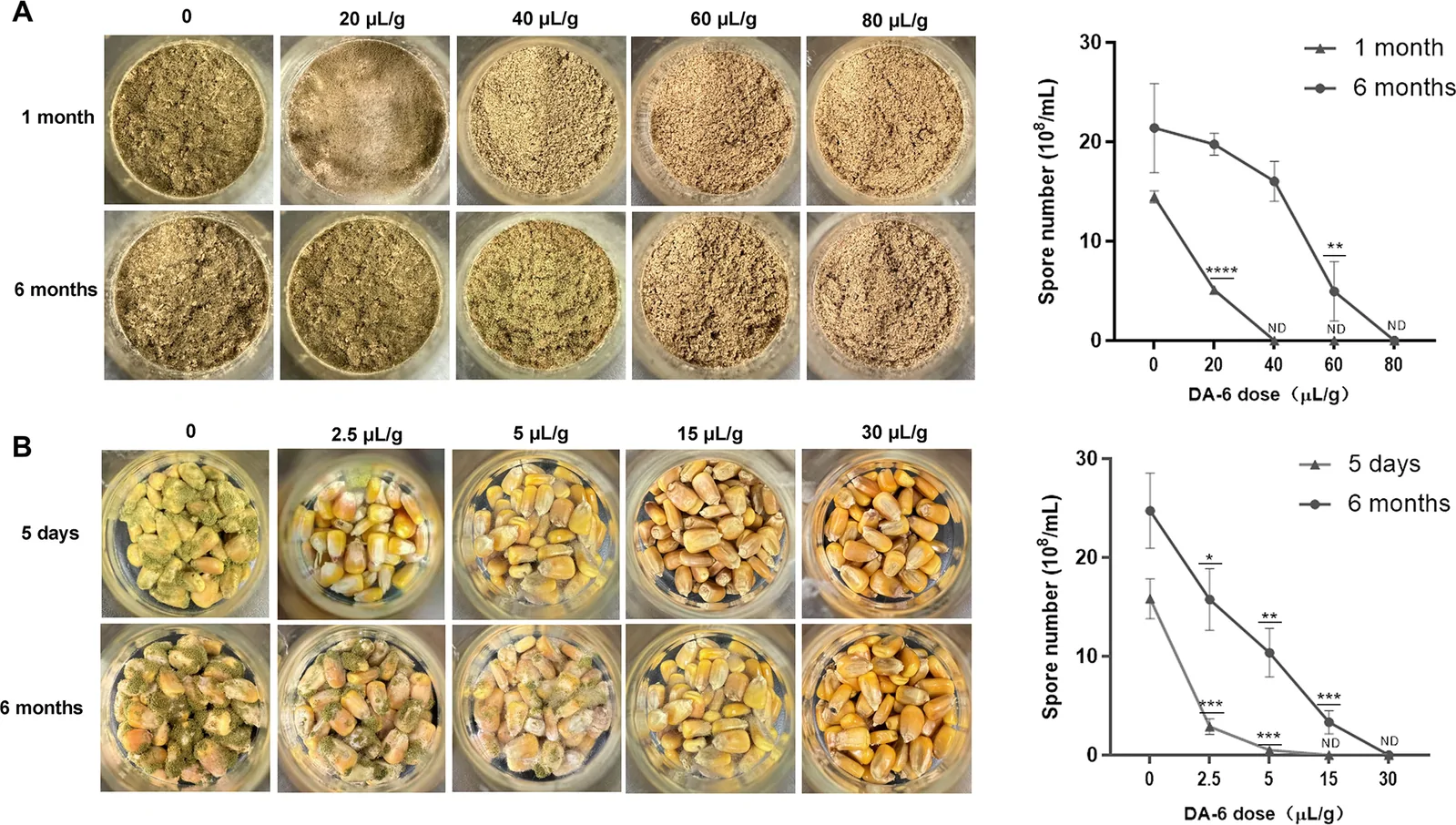
Effect of DA-6 on *A. flavus* infection in rice bran and maize. (**A**) Growth and sporulation of *A. flavus* on rice bran treated with different concentrations of DA-6 after 1 and 6 months of storage, spore counts are shown on the right. (**B**) Visual appearance of *A. flavus* infection on maize kernels treated with DA-6 at different concentrations, with inhibition rates of sporulation calculated relative to the control. Data are shown as mean ± SD, *n* = 3. *P < 0.05, **P < 0.01, ***P < 0.001, ****P < 0.0001; ND, not detected.

The inhibitory effects of DA-6 on *A. flavus* infection were further assessed in rice bran and maize. As shown in Fig. 2A, abundant sporulation occurred in the control group, whereas DA-6 treatment markedly reduced conidia production in a dose-dependent manner. Both 60 μL/g and 80 μL/g (corresponding to approximately 54.4 and 72.6 mg/g active ingredient, respectively) almost completely suppressed sporulation after 1 month. After 6 months of storage, complete suppression was maintained at 80 μL/g, whereas 60 μL/g still showed substantial but incomplete inhibition. A similar dose-dependent inhibitory pattern was observed on maize kernels (Fig. 2B). After 6 months, inhibition exceeded 85% at ≥15 µL/g and 30 μL/g maintained full inhibition. These findings indicate that DA-6 holds strong potential as an effective agent to protect seed crops from *A. flavus* contamination.

### DA-6 induces plasma membrane disruption and cytoplasmic leakage in *A. flavus*

**Fig 3:**
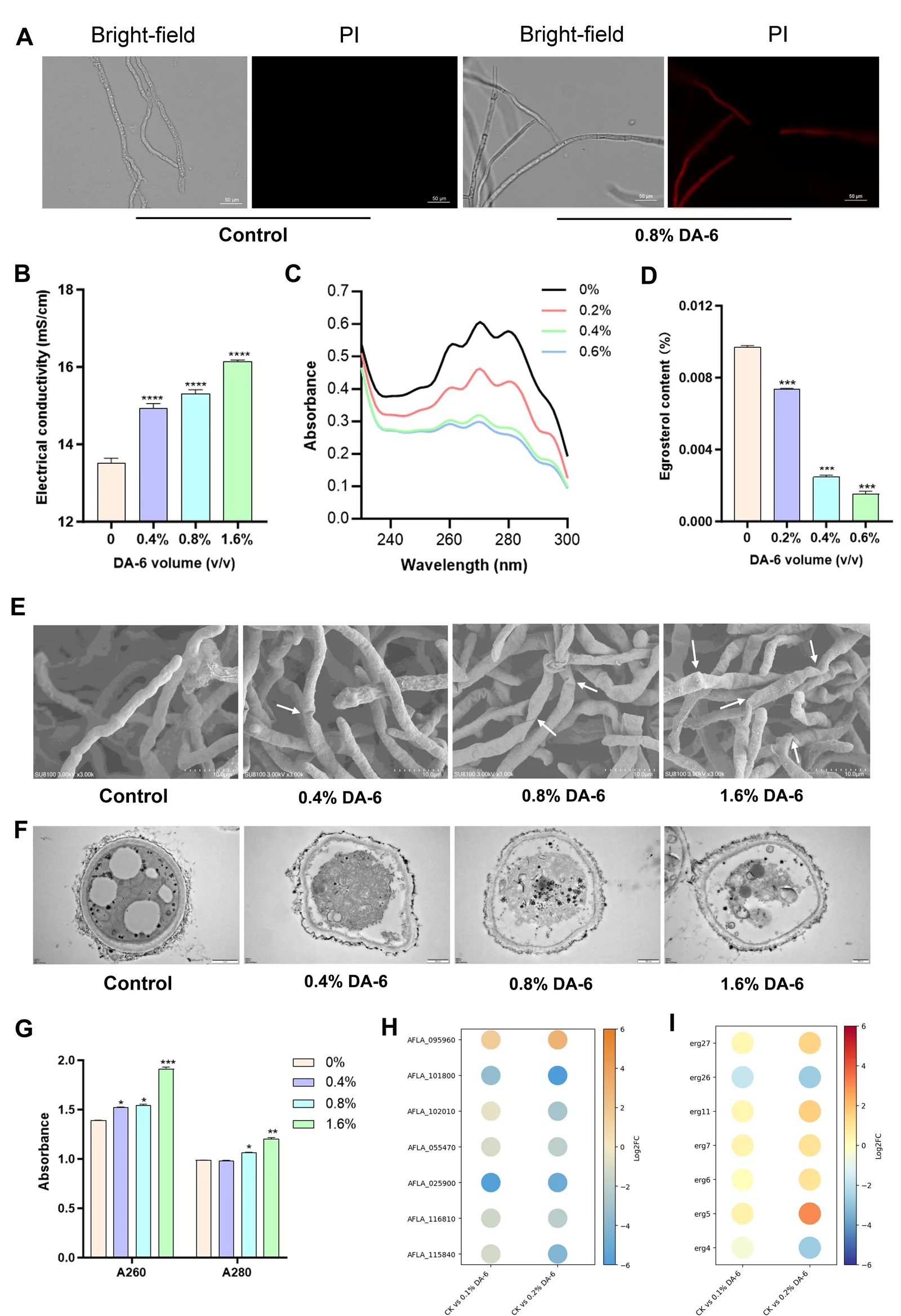
Effect of DA-6 on the plasma membrane integrity of *A. flavus*. (**A**) Fluorescence microscopy images of hyphae stained with PI. (**B**) Changes in extracellular conductivity of cultures in response to DA-6 treatment. (**C**) Ultraviolet absorption spectra of ergosterol extracted from *A. flavus* mycelia treated with different concentrations of DA-6. (**D**) Quantitative analysis of ergosterol content in *A. flavus* mycelia following treatment with different concentrations of DA-6. (**E**) SEM images of *A. flavus* hyphae treated with DA-6. The white arrows pointed out the deformed and the abnormal hyphae. (**F**) TEM images of hyphae treated with DA-6. (**G**) Absorbance at *A*_260_ and *A*_280_ nm of culture supernatants. (**H** and **I**) Heatmap of differentially expressed genes (DEGs) related to chitin and ergosterol synthesis after DA-6 treatment. The asterisk indicates significant differences, *P < 0.05, **P < 0.01, ***P < 0.001, ****P < 0.0001.

To investigate whether DA-6 affects the membrane integrity of *A. flavus*, hyphae were examined using propidium iodide (PI) staining. Fluorescence microscopy showed strong red PI signals in DA-6-treated hyphae, whereas only weak signals were observed in the control, indicating increased membrane permeability (Fig. 3A). Consistently, extracellular conductivity was significantly elevated and continued to increase over time, reflecting electrolyte leakage (Fig. 3B), which was further supported by increased A260 and A280 values (Fig. 3G), indicating leakage of intracellular nucleic acids and proteins. Moreover, ergosterol content in *A. flavus* mycelia was markedly reduced by DA-6, suggesting disruption of membrane composition and stability (Fig. 3C and D). Further scanning electron microscopy (SEM) images showed that control hyphae were smooth and intact, while DA-6-treated hyphae displayed collapsed, irregular, and disrupted surfaces (Fig. 3E). Collectively, these results demonstrate that DA-6 compromises plasma membrane integrity of *A. flavus*.

Transmission electron microscopy provided more detailed evidence of ultrastructural damage (Fig. 3F). Control hyphae exhibited intact cell walls and membranes, uniform cytoplasm, and clear organelles. In contrast, DA-6 treatment induced progressive alterations, including loosened cell walls, vacuolation, and disorganized cytoplasm. The cellular architecture was almost completely destroyed by 1.6% DA-6 treatment, with ruptured cell walls, collapse of membrane structures, and leakage of cytoplasmic contents. These findings corroborate the ultrastructural observations and confirm that DA-6 compromises membrane integrity, leading to leakage of intracellular contents.

Transcriptomic analysis revealed that many membrane-related genes were downregulated after DA-6 treatments. DEGs were mainly enriched in the gene ontology (GO) term “membrane” within the cellular component (CC) category, with 401 and 590 genes downregulated, as well as 240 and 292 genes upregulated in the 0.1% and 0.2% DA-6 groups, respectively (Fig. S2A and B). Notably, several cell wall biosynthesis genes, including chitin synthases (AFLA_101800, AFLA_055470, AFLA_025900), a glucan synthase (AFLA_116810), and a GPI-anchored protein (AFLA_115840), showed reduced transcript levels in both DA-6 treatment groups (Fig. 3H). Several DEGs involved in ergosterol biosynthesis were also identified, most of which were upregulated, despite the experimentally observed reduction in ergosterol content, possibly reflecting an adaptive response to membrane stress induced by DA-6 treatment (Fig. 3I).

### DA-6 triggers ROS accumulation and modulates antioxidant enzyme activities in *A. flavus*

**Fig 4:**
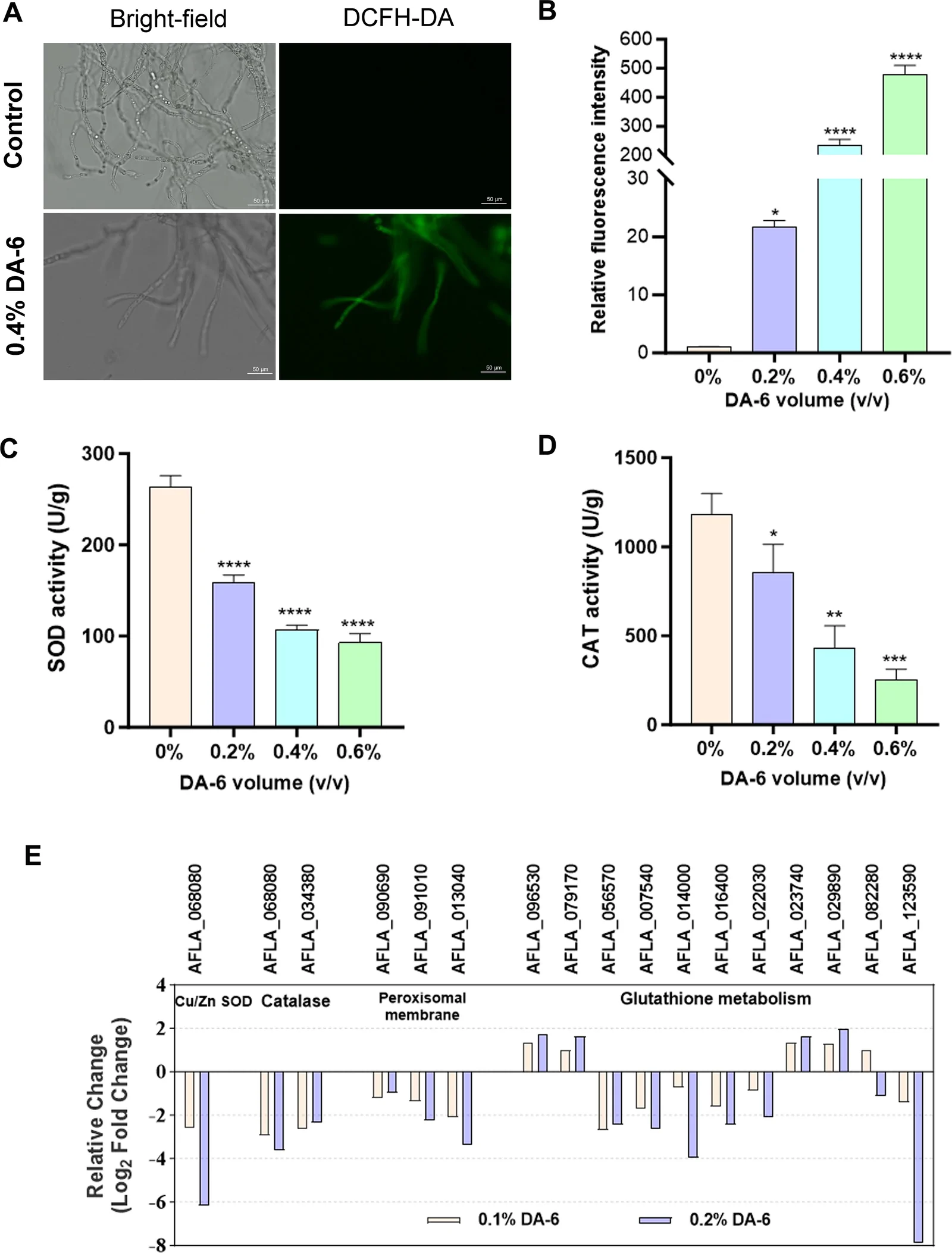
Effect of DA-6 on ROS accumulation and antioxidant enzyme activities in *A. flavus*. (**A**) Fluorescence microscopy images of hyphae stained with DCFH-DA showing ROS distribution. (**B**) Quantification of fluorescence intensity. (**C**) Superoxide dismutase (SOD) activity. (**D**) Catalase (CAT) activity. (**E**) Transcriptome expression profiles of DEGs (*P* < 0.05, |log_2_ FC| ≥ 1) related to catalase and glutathione metabolism in *A. flavus* after DA-6 treatments. The asterisk indicates significant differences, *P < 0.05, **P < 0.01, ***P < 0.001, ****P < 0.0001.

DA-6 treatment markedly increased intracellular ROS in *A. flavus*. Hyphae stained with DCFH-DA, a fluorescent probe widely used for detecting intracellular ROS accumulation, showed much stronger fluorescence after DA-6 exposure than the control (Fig. 4A), and quantitative analysis confirmed a significant, concentration-dependent rise in ROS levels (Fig. 4B). Meanwhile, the measured activities of antioxidant enzyme exhibited opposite trends. Both superoxide dismutase and CAT activities gradually declined with increasing DA-6 concentrations (Fig. 4C and D). At the transcriptome level, several antioxidant-related genes were downregulated following DA-6 treatment, including superoxide dismutase (Cu/Zn SOD, AFLA_068080), catalase (AFLA_034380, AFLA_090690), peroxisomal membrane protein genes (AFLA_091010, AFLA_013040, AFLA_096530), and multiple genes annotated to glutathione metabolism (Fig. 4E). Together, these data indicate that DA-6 exposure is associated with oxidative stress in *A. flavus*, characterized by ROS accumulation, and impaired antioxidant capacity.

### DA-6 disrupts MMP and ATP content in *A. flavus*

**Fig 5:**
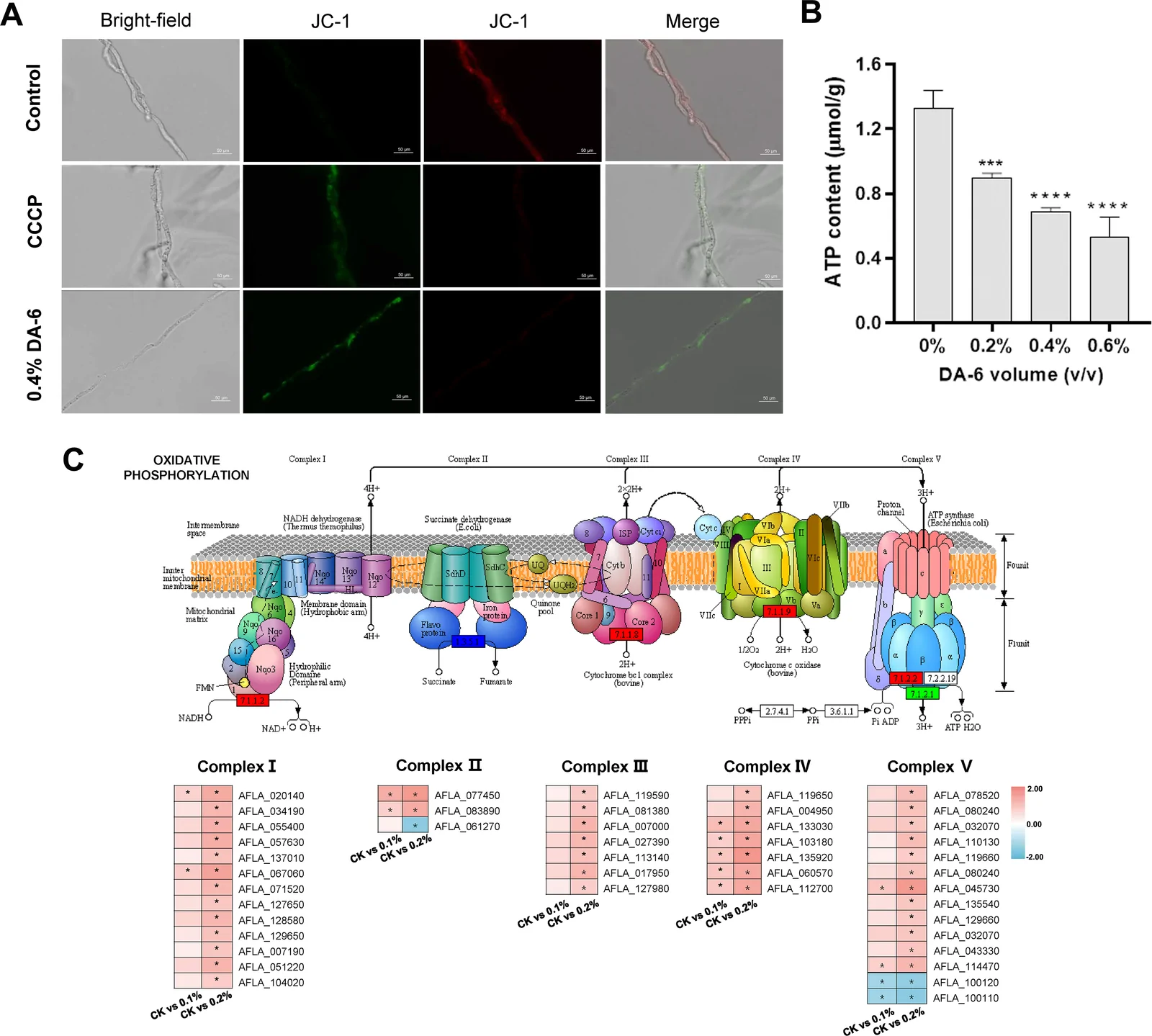
Effect of DA-6 on the MMP and intracellular ATP content of *A. flavus.* (**A**) Membrane potential (MMP) changes visualized by JC-1 staining of *A. flavus* mycelia. Control hyphae exhibited strong red fluorescence, whereas CCCP treatment (positive control) and 0.4% DA-6 treatment showed green fluorescence. (**B**) Intracellular ATP levels of *A. flavus* mycelia treated with different concentrations of DA-6. The asterisk indicates significant differences, ***P < 0.001, ****P < 0.0001. (**C**) Heatmaps of log_2_ FC values of the DEGs enriched in oxidative phosphorylation (ko00190). The map was developed in the KEGG pathway database. The asterisk indicates DEGs with a log_2_FC > 1 and FDR < 0.05.

JC-1 staining was performed to examine the effect of DA-6 on *A. flavus* mitochondria. The control group mainly emitted red fluorescence, indicating a high mitochondrial membrane potential (MMP), whereas the hyphae treated with 0.4% DA-6 exhibited green fluorescence, indicating a reduction in MMP and suggesting mitochondrial dysfunction (Fig. 5A). In addition, DA-6 treatment caused a concentration-dependent decline in intracellular ATP content, and the reduction reached 60.2% at 0.6% DA-6 (Fig. 5B).

To probe mitochondrial involvement, we examined transcriptional changes in energy metabolism pathways. KEGG enrichment showed significant representation of oxidative phosphorylation pathway following DA-6 treatment (Fig. S2C and D). Accordingly, many core subunits of the electron transport chain complexes I–V, including NADH dehydrogenase, succinate dehydrogenase, cytochrome *c* oxidase, and ATP synthase, were predominantly upregulated after DA-6 exposure (Fig. 5B). Beyond this pathway, GO analysis identified 60 additional mitochondrial DEGs induced by 0.2% DA-6 exposure (Table S3), encompassing mitochondrial ribosomal proteins, TIM/TOM import components (e.g., Tim10/Tim13), cytochrome-*c* oxidase assembly factors, and mitochondrial carrier proteins. Most of these genes also showed transcriptional upregulation, indicating a broad mitochondrial response to DA-6 treatment that may reflect a compensatory adaptation to mitochondrial stress. These results suggest that DA-6 profoundly influences mitochondrial electron transport and energy metabolism.

### DNA damage and autophagy induced by DA-6 treatment

**Fig 6:**
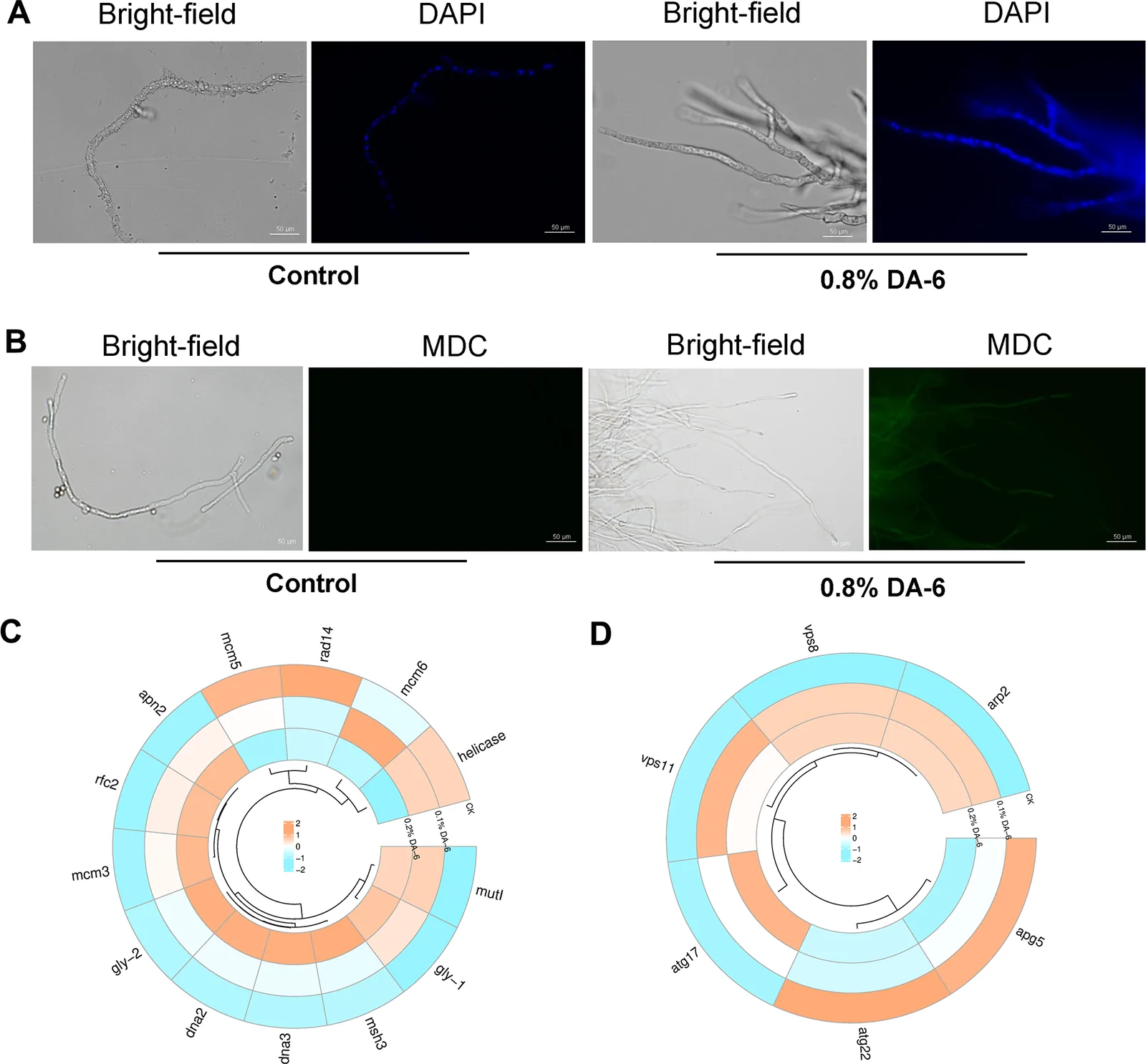
DNA damage and autophagy induced by DA-6 treatment. Fluorescence microscopy analysis of DNA damage (**A**) and autophagy (**B**) induced by DA-6 in *A. flavus. A. flavus* mycelia were treated with 0.8% DA-6 and stained with DAPI for nuclear damage and MDC for autophagy detection. Increased fluorescence intensity was observed with higher DA-6 concentrations, indicating enhanced DNA damage and autophagy. (**C** and **D**) Heatmaps of DEGs related to chitin and ergosterol biosynthesis.

To assess whether DA-6 induced nuclear damage and autophagy in *A. flavus*, we performed DAPI and MDC staining. As shown in Fig. 6A, control mycelia displayed weak blue fluorescence, whereas DA-6-treated mycelia caused markedly enhanced nuclear fluorescence, suggesting nuclear abnormalities and chromatin condensation. These changes are commonly associated with nuclear stress and may reflect DNA damage-related responses. MDC staining revealed weak fluorescence in control mycelia, while bright green puncta appeared after DA-6 exposure, corresponding to the accumulation of autophagic vacuoles (Fig. 6B). These results suggest that DA-6 induces nuclear alterations and autophagy in *A. flavus* cells.

Transcriptomic analysis further supported these findings (Fig. 6C and D). Most genes associated with DNA replication and repair—including *mcm3*, *mutI*, *msh3*, *dna2*, and DNA glycosylase—were upregulated under DA-6 treatment, suggesting the activation of a compensatory DNA repair response. Additionally, several autophagy-related genes (*apg5*, *atg22*, *apg17*, *vps11*, *vps8*) showed transcriptional changes, indicating that the autophagic process was also affected by DA-6.

### DA-6 suppresses aflatoxin biosynthesis and perturbs transcriptional regulatory networks

**Fig 7:**
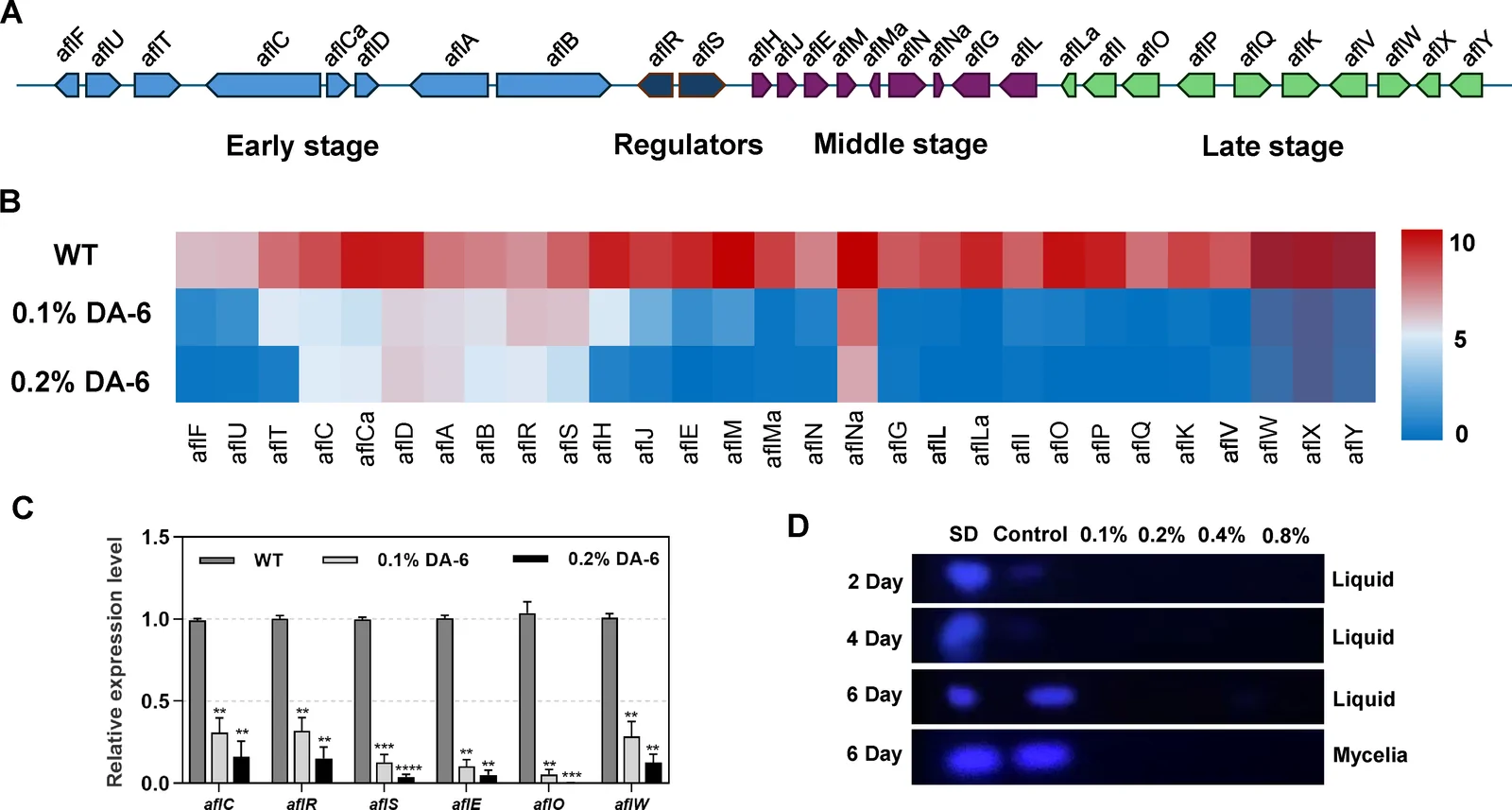
DA-6 suppresses *A. flavus* aflatoxin biosynthetic gene cluster expression and AFB₁ production. (**A**) Genetic organization of the aflatoxin biosynthetic cluster. (**B**) Heatmap of cluster gene expression levels in WT, 0.1%, and 0.2% DA-6 groups based on transcriptomic data. Color scale indicates log₂ (FPKM + 1) values. (**C**) qRT-PCR validation of selected AF cluster genes. (**D**) TLC analysis of AFB₁ production under different DA-6 concentrations in liquid cultures and mycelial extracts. Data are presented as mean ± SD (*n* = 3). Statistical analysis was performed using one-way ANOVA followed by Dunnett’s test for comparison with the WT group. **P < 0.01, ***P < 0.001, ****P < 0.0001.

DA-6 treatment almost completely abolished AFB₁ biosynthesis in *A. flavus*. TLC analysis of both culture filtrates and mycelial extracts revealed no detectable toxin bands in any DA-6-treated group, even at the lowest concentration (0.1%) (Fig. 7D).

Transcriptomic KEGG enrichment further revealed that the aflatoxin biosynthesis pathway (ko00254) ranked first among all significantly enriched pathways in both 0.1% and 0.2% DA-6 treatments, indicating that DA-6 primarily targets aflatoxin metabolism (Fig. S2C and D). As shown in Fig. 7A, the aflatoxin biosynthetic cluster in *A. flavus* comprises approximately 30 genes that function sequentially in early, middle, and late stages of aflatoxin biosynthetic pathway. Transcriptome analysis demonstrated that DA-6 treatment led to extensive transcriptional repression across the entire cluster (Fig. 7B). All 30 cluster genes were significantly downregulated under both DA-6 treatments, with large |log₂ FC| values (Table S4). Early-stage genes (*aflF*, *afU*, *aflT*) were markedly reduced, whereas several middle to late structural genes (*aflE*, *aflL*, *aflLa*, *aflO*, *aflP*, *aflQ, aflK, aflV*) showed extremely low expression levels, with FPKM values equal to zero in the RNA-seq data set, indicating undetectable expression under DA-6 treatment. Moreover, the cluster-specific transcriptional activators *aflR* and *aflS* were both significantly downregulated, with *aflR* exhibiting log₂ FCs of –1.81 and –3.72 under 0.1% and 0.2% DA-6 treatments, respectively. Six representative AF cluster genes, including early (*aflF*), middle (*aflL*), and late (*aflP*) structural genes as well as the regulatory genes *aflR* and *aflS*, were selected for qPCR validation, showing expression trends consistent with the transcriptome data (Fig. 7C).

Aflatoxin biosynthesis in *A. flavus* is regulated not only by cluster genes but also by multiple upstream transcriptional regulators. A total of 108 transcription factors (TFs) were significantly affected following DA-6 treatment (|Log₂ FC| ≥ 1, padj < 0.05) (Table S5). Several regulators associated with fungal development and secondary metabolism, including BrlA, FlbC, SteA, StuA, CreA, MtfA, and NosA, were transcriptionally repressed.

**Fig 8:**
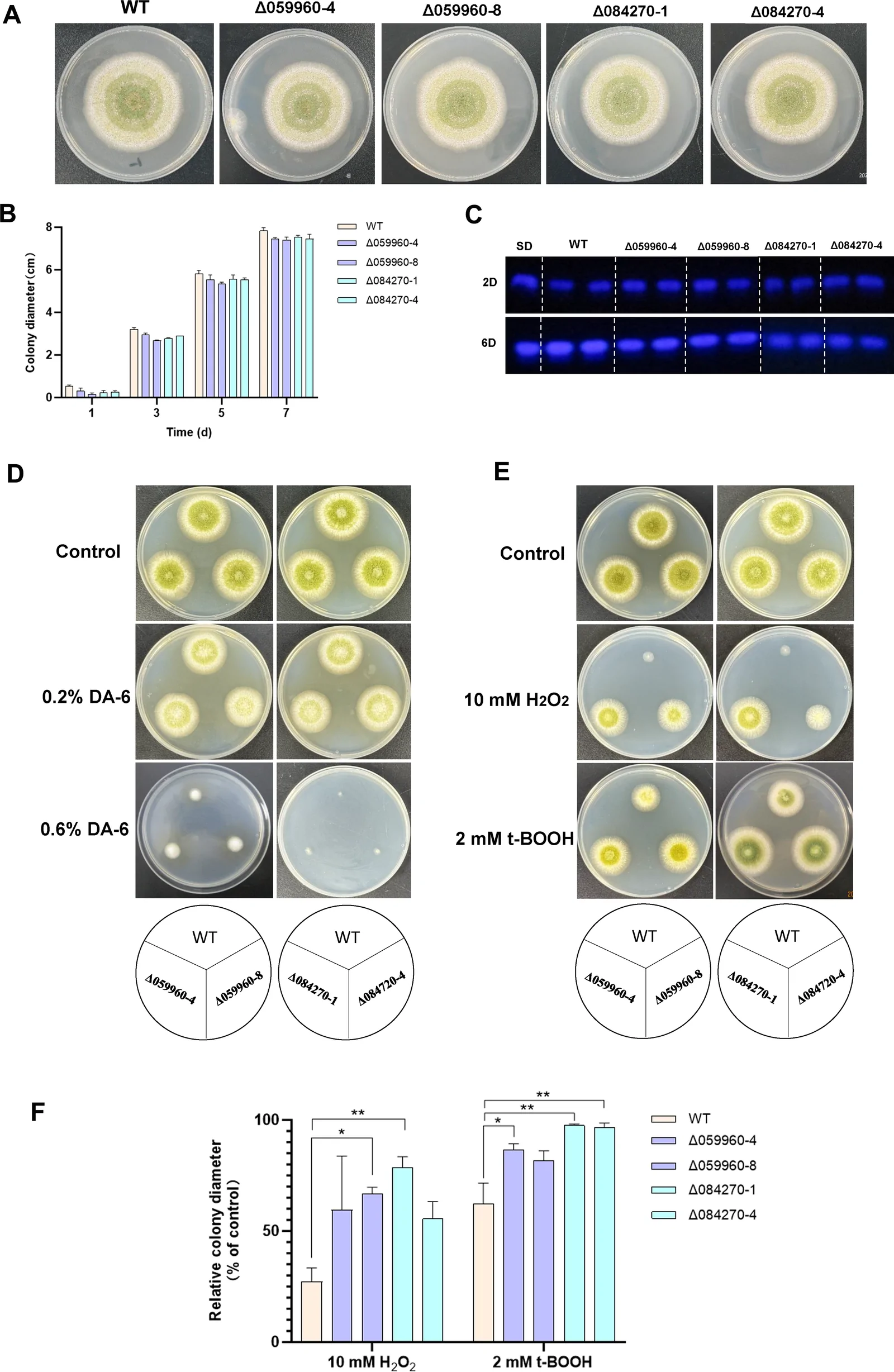
Functional characterization of two Zn_2_Cys_6_ transcription factor (AFLA_059960 and AFLA_084720) deletion mutants in growth, aflatoxin production, and oxidative stress response. (**A**) Colony morphology of WT and transcription factor deletion mutants grown on PDA plates for the indicated time. (**B**) Colony diameter measurements of WT and mutant strains during cultivation. Data represent mean ± SD. (**C**) Thin-layer chromatography (TLC) analysis of aflatoxin production by WT and mutant strains at 2 and 6 days of culture. (**D**) Growth phenotypes of WT and mutants under DA-6 treatment (0.2% and 0.6%) compared with control conditions. (**E**) Sensitivity assays of WT and mutants under oxidative stress induced by 10 mM H₂O₂ and 2 mM tert-butyl hydroperoxide (t-BOOH). (**F**) Quantification of relative colony diameter under oxidative stress conditions shown in panel E. Values are expressed relative to untreated controls (mean ± SD). Asterisks indicate statistically significant differences (*P < 0.05, **P < 0.01).

In addition to these known regulators, several uncharacterized Zn₂Cys₆-type TFs also exhibited pronounced transcriptional responses to DA-6 treatment. Among them, AFLA_059960 and AFLA_084720 exhibited the most significant repression and were, therefore, selected for functional characterization. The deletion of these TFs, which was confirmed by diagnostic PCR and RT-qPCR validation (Fig. S3B through D), did not significantly affect fungal growth, aflatoxin production, or DA-6 sensitivity (Fig. 8A through C). However, both mutants displayed enhanced tolerance to oxidative stress (Fig. 8D through F), suggesting that these TFs may participate in cellular redox regulation rather than directly controlling aflatoxin production. This observation suggests that DA-6 may disrupt fungal adaptation capacity to oxidative stress by modulating specific transcriptional regulators associated with redox homeostasis.

## DISCUSSION

Contamination of food and feed with *A. flavus* and aflatoxins poses a serious threat to food safety and public health worldwide. Due to the increasing restrictions on synthetic fungicides and the emergence of resistant fungal strains, there is an urgent need to explore safe and environmentally friendly antifungal agents. Recently, attention has turned to naturally derived or physiologically active compounds for fungal control. Certain plant growth regulators (PGRs), such as cytokinin, were shown to directly inhibit the growth, development, and virulence of fungal phytopathogens of *Botrytis cinerea* by targeting the cytoskeleton and cellular trafficking (10). In addition, indole-3-acetic acid (IAA) has been reported to inhibit bacterial pathogens like *Pseudomonas aeruginosa* by disrupting biofilm formation and reducing cell viability (32). However, the antifungal potential of PGRs has remained largely unexplored, and no studies have evaluated their effects on *A. flavus* or aflatoxin biosynthesis.

In this study, we for the first time identified DA-6, a commonly used plant growth regulator, as a potent inhibitor of *A. flavus* growth and aflatoxin biosynthesis. Among eight representative plant growth regulators tested, DA-6 exhibited potent antifungal activity, with a MIC of 0.8% (vol/vol) (Fig. 1A). This inhibitory potency is comparable to several reported plant-derived antifungal compounds such as methyl jasmonate (MeJA), cuminaldehyde, and *p*-anisaldehyde (13, 24, 25). DA-6 significantly suppressed colony expansion and spore germination of *A. flavus* in a dose-dependent manner (Fig. 1B), and effectively reduced AFB₁ accumulation (Fig. 7D). Furthermore, DA-6 treatment markedly suppressed fungal colonization, as evidenced by the substantial reduction in conidial production during maize and rice bran storage (Fig. 2), highlighting its potential for controlling *A. flavus* contamination in stored agricultural commodities. As an agricultural plant growth regulator with established applications and a favorable safety profile (33), DA-6 may represent a multifunctional compound for further development in fungal contamination management.

The lipophilic nature of DA-6 suggested that the cytoplasmic membrane, a common target for many antifungal agents (23, 34, 35), may be its primary site of action. The integrity of the cytoplasmic membrane is critical for maintaining fungal viability. Several plant-derived antifungal compounds, including cuminaldehyde, 1-nonanol, 1-octanol, and honokiol, have been shown to disrupt the cell membrane integrity of *A. flavus*, leading to cytoplasmic leakage and loss of viability (23, 25, 36, 37). Consistent with these findings, our results demonstrated that DA-6 significantly increased membrane permeability, as evidenced by PI stain and the dose-dependent increase in extracellular conductivity (Fig. 3A and B). This membrane destabilization was further corroborated by SEM and TEM analyses showing cell surface collapse, membrane rupture, and cytoplasmic disintegration (Fig. 3E and F). Such damage was accompanied by a notable reduction in ergosterol content (Fig. 3C and D), a key sterol maintaining membrane fluidity and structural integrity (38). Since ergosterol depletion compromises membrane stability and function (39, 40), these findings collectively suggest that DA-6 exerts its antifungal activity by disrupts membrane composition and integrity, ultimately leading to cytoplasmic leakage and cell lysis.

Transcriptomic analysis further supported the membrane-targeting effects of DA-6. DEGs were significantly enriched in the GO terms “membrane,” with hundreds of genes downregulated after DA-6 exposure (Fig. S2). Several genes involved in ergosterol biosynthesis, including *erg5* (AFLA_028640), *erg6* (AFLA_041230), *erg7* (AFLA_006860), and *erg11* (AFLA_131060), were moderately upregulated under 0.2% DA-6 treatment, whereas *erg4* (AFLA_030250) and *erg26* (AFLA_058900) were markedly downregulated, indicating that DA-6 disturbs sterol biosynthetic balance rather than uniformly repressing the pathway. Moreover, the newly synthesized ergosterol may not be efficiently incorporated into severely damaged membranes, which could also contribute to the reduced ergosterol content observed after DA-6 treatment. In addition, cell wall-related genes were also strongly affected. Chitin is a key structural component of fungal cell walls that contributes to cell wall rigidity and integrity (41, 42). Several genes associated with chitin biosynthesis and cell wall remodeling exhibited significant transcriptional changes following DA-6 treatment. Specifically, AFLA_095960 encoding the chitin synthase D was slightly upregulated, while most chitin-related genes, including chitinase (AFLA_101800, AFLA_102010), chitin synthase (AFLA_055470), and chitin deacetylase (AFLA_025900), were strongly downregulated, together with alpha-1,3-glucan synthase (AFLA_116810) and GPI-anchored protein (AFLA_115840). This suggests that DA-6 may interfere with chitin-associated cell wall remodeling and cell wall biogenesis. Similar transcriptional responses were reported in *A. flavus* exposed to honokiol, where multiple cell wall-related genes were downregulated (37) . In contrast, 1-nonanol induced compensatory upregulation of both synthase and hydrolase genes (36). These differences suggest that DA-6 may primarily disrupt cell wall homeostasis rather than triggering a predominant compensatory repair response.

In eukaryotic cells, ROS are by-products of normal oxygen metabolism and also function as important signaling molecules. However, excessive ROS accumulation can disrupt cellular redox homeostasis and cause oxidative damage to cellular components, particularly membrane lipids and proteins (43). Therefore, the balance between ROS generation and antioxidant defenses is, therefore, crucial for maintaining cellular stability (44, 45). In this study, DA-6 treatment markedly disturbed the redox homeostasis in *A. flavus*, as evidenced by ROS accumulation and reduced antioxidant enzyme activities (Fig. 4). This pattern was consistent with transcriptomic data, in which genes encoding superoxide dismutase (Cu/Zn SOD, AFLA_068080), and catalase (AFLA_034380 and AFLA_090690) were significantly downregulated in both DA-6 treatments (Fig. 4). Similar trends have been observed in *A. flavus* exposed to L-cysteine hydrochloride (L-CH) and *p*-anisaldehyde (24, 46). However, other antifungal agents such as 1-nonanol and linalool have been reported to trigger elevated SOD and CAT activities as a compensatory antioxidant response (34, 36). The observed discrepancy may arise from differences in chemical properties and cellular targets among these compounds. Therefore, we infer that DA-6 exposure leads to impaired oxidative defense capacity in *A. flavus*, resulting in excessive ROS accumulation and subsequent oxidative stress.

Mitochondria are the main source and also a primary target of ROS in eukaryotic cells. Excessive ROS can damage mitochondrial membranes and mitochondrial DNA, leading to impaired oxidative phosphorylation, loss of MMP, and decreased ATP synthesis (47–49). Consistent with this, DA-6 treatment caused a marked ROS accumulation accompanied by a decline in MMP and cellular ATP (Fig. 5A and B), suggesting that depolarization of the mitochondrial membrane impaired oxidative phosphorylation and energy production. Paradoxically, transcriptomic analysis revealed a broad transcriptional upregulation of oxidative phosphorylation and other mitochondrion-associated genes following DA-6 exposure (Fig. 5C; Table S3). This pattern contrasts with previous studies in *A. flavus* (34, 50) and *Ustilaginoidea virens* (51), where antifungal treatments caused repression of mitochondrial respiratory genes. Mitochondria, as essential membrane-bound organelles hosting numerous metabolic processes, have been recognized as key cellular targets of many essential oils and natural compounds (52–54). We speculated that the upregulation of electron transport chain-related genes observed here may represent an early or compensatory response of *A. flavus* to maintain energy homeostasis and re-establish redox balance under DA-6-induced oxidative stress. Similar compensatory mitochondrial activation has been reported in fungi experiencing moderate oxidative or metabolic stress, suggesting a self-protective adjustment (25, 55, 56).

Severe oxidative and energetic stress can compromise genomic integrity and trigger autophagy as either a protective or degenerative response in fungi (57, 58). In this study, DA-6 treatment induced both DNA damage and autophagy in *A. flavus* mycelia (Fig. 6). Transcriptomic data further supported these observations, showing significant upregulation of genes involved in DNA replication (*dna2*, AFLA_050550/050540, *rfc2*, AFLA_086730), and mismatch or nucleotide excision repair (*mutI*, AFLA_078200; *msh3*, AFLA_073760; AP endonuclease 2, AFLA_103190; DNA glycosylases, AFLA_033610/AFLA_074990; DNA helicase, AFLA_087500), suggesting activation of compensatory DNA repair mechanisms. Minichromosome maintenance (MCM) proteins are essential for proper initiation and control of DNA replication (59). In this study, multiple MCM3 homologs (AFLA_073390, AFLA_116920, AFLA_093540) showed mixed regulation under DA-6 treatment, which may reflect a cellular attempt to balance replication-fork recovery with suppression of global DNA synthesis under stress. Autophagy is a conserved adaptive mechanism frequently triggered under environmental or oxidative stress to remove damaged cellular components and restore metabolic balance (60, 61). In fungi, moderate activation of autophagy generally supports cell survival by maintaining homeostasis, whereas excessive or prolonged activation may become detrimental and lead to self-degradation and cell death (62). In parallel, autophagy-related genes (*Apg5*, *Atg22*, *Apg17*, *Vps11, Vps8*) exhibited transcriptional changes, consistent with the activation of the autophagic process. Similar activation of DNA repair and autophagy has been observed in *A. flavus* conidia exposed to linalool (34), suggesting that these responses represent a conserved defense strategy against oxidative damage.

Fungal secondary metabolism is closely coupled with cellular redox and energy states (63–65). Under environmental or metabolic stress, fungi often undergo global transcriptional and metabolic reprogramming to maintain homeostasis (66). In this study, DA-6 exhibited a strong inhibitory effect on aflatoxin biosynthesis, even at concentrations that caused no significant reduction in mycelial biomass (Fig. 1C and 7B). Such complete transcriptional silencing of the AF gene cluster under low-dose DA-6 treatment (0.1% and 0.2%) suggests that DA-6 may suppress energetically costly secondary metabolic pathways as a self-protective strategy. Compared with other reported inhibitors such as L-CH or MeJA, which mainly downregulate middle- or late-stage AF structural genes while partially retaining early-stage transcription (13, 46), DA-6 caused broad repression across the entire cluster, including the pathway-specific activators *aflR* and *aflS*. The nearly complete loss of transcription for late-stage genes (*aflE*, *aflL*, *aflLa*, *aflO*, *aflP*, *aflQ, aflK, aflV*), whose FPKMs were close to zero indicates that the biosynthetic process was effectively halted consistent with the absence of detectable AFB₁ in both culture filtrates and mycelia (Fig. 7D).

Beyond the AF cluster, DA-6 also markedly downregulated several global regulatory genes such as *laeA* and *veA*, the core members of the velvet complex that coordinate fungal development and secondary metabolism (1, 31, 67). LaeA, in particular, functions as a methyltransferase-like regulator that modulates chromatin accessibility of multiple secondary metabolite gene clusters, and its suppression is frequently associated with transcriptional silencing of *aflR* and inhibition of AF production (67, 68). The concurrent repression of LaeA, VeA, and additional transcriptional regulators, such as BrlA, FlbC, StuA, CreA, and MtfA, all of which play important roles in fungal development and secondary metabolism, suggests that DA-6 interferes with higher-order regulatory circuits governing morphogenesis and metabolic coordination. We speculate that DA-6 perturbs intracellular signaling homeostasis, particularly redox balance, thereby triggering widespread transcriptional reprogramming associated with developmental and metabolic control. Such interference could suppress both *aflR/aflS*-dependent activation and the broader secondary metabolism machinery, ultimately leading to the near-complete shutdown of aflatoxin biosynthesis.

Notably, transcriptomic analysis also identified numerous previously uncharacterized transcription factors exhibiting strong transcriptional responses to DA-6 exposure. Functional characterization of two representative Zn₂Cys₆-type TFs, AFLA_059960 and AFLA_084720, revealed that their deletion did not significantly affect fungal growth or aflatoxin production (Fig. 8A through C). However, both mutants displayed enhanced tolerance to oxidative stress, suggesting that these TFs may primarily participate in cellular redox adaptation rather than directly regulating aflatoxin biosynthesis (Fig. 8D through F). These findings indicate that DA-6 may suppress fungal fitness by perturbing multiple transcriptional regulatory pathways involved in oxidative stress adaptation, rather than acting through a single dedicated regulatory node.

### Conclusion

**Fig 9:**
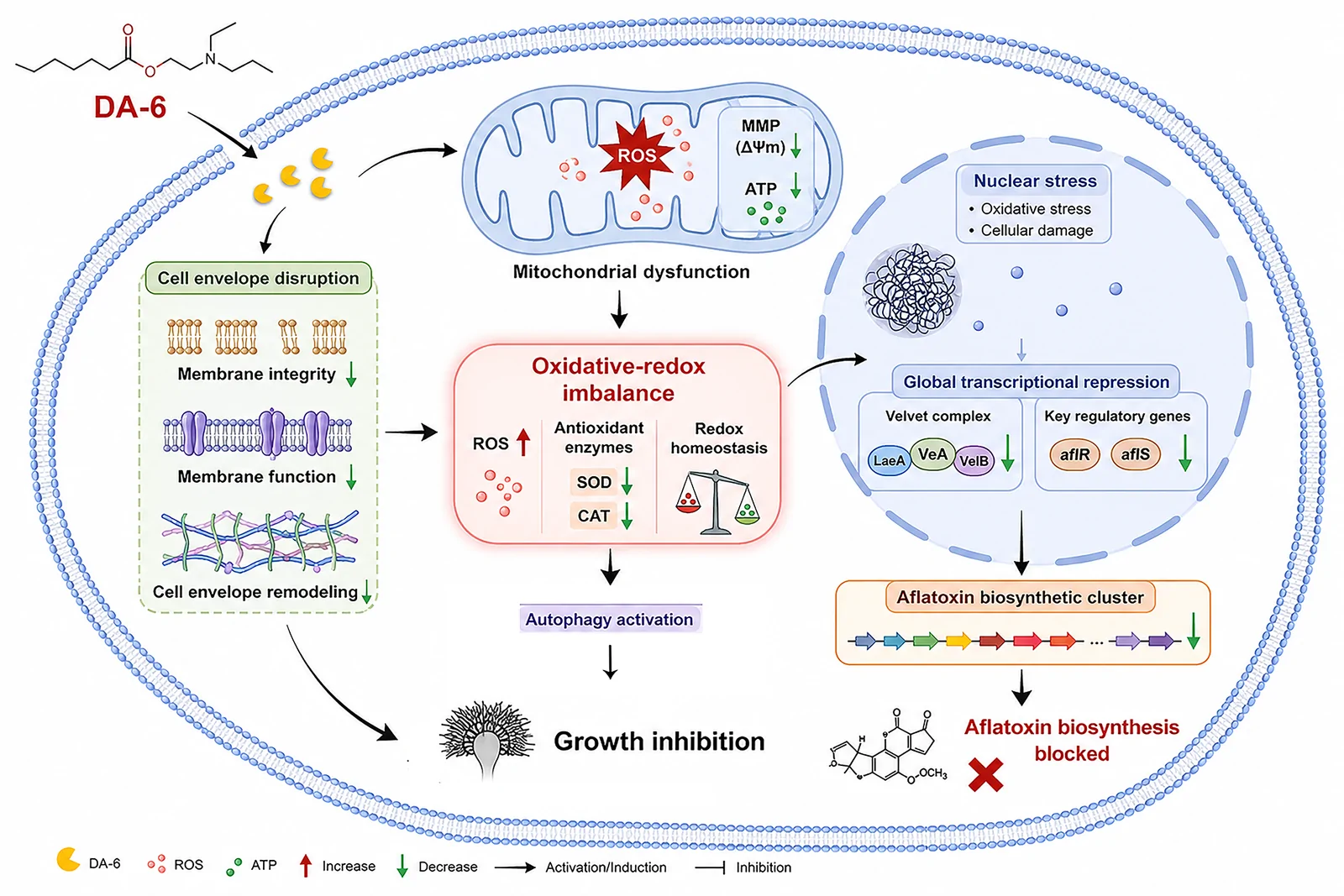
Proposed model of antifungal effect of DA-6 on *Aspergillus flavus*. DA-6 induces membrane disruption and oxidative imbalance, leading to mitochondrial dysfunction, ATP depletion, DNA damage, and autophagy. These cellular stresses collectively repress developmental and secondary metabolism regulators (LaeA/VeA, AflR/AflS), resulting in the global shutdown of aflatoxin biosynthesis. DA-6, diethyl aminoethyl hexanoate; ROS, reactive oxygen species; SOD, superoxide dismutase; CAT, catalase.

In conclusion, this study demonstrates for the first time that the widely used plant growth regulator DA-6 exhibits antifungal activity against *Aspergillus flavus* growth and effectively suppresses aflatoxin biosynthesis. Integrating physiological evidence with transcriptomic analysis, we propose a mechanistic model describing the antifungal action of DA-6 (Fig. 9). DA-6 primarily disrupts plasma membrane integrity and mitochondrial function, resulting in excessive ROS accumulation, mitochondrial depolarization, and reduced ATP production. These findings collectively indicate that disruption of oxidative-redox homeostasis represents a central event underlying the antifungal activity of DA-6.

At the transcriptional level, DA-6 broadly repressed global regulatory genes such as *laeA* and *veA*, together with the key aflatoxin regulatory genes (*aflR*, *aflS*), leading to a near-complete transcriptional silencing of the AF biosynthetic gene cluster. These findings indicate that DA-6 interferes with both global developmental regulation and toxin biosynthesis programs in *A. flavus*. Functional analysis of two highly DA-6-responsive Zn₂Cys₆ transcription factors further suggested the involvement of redox-associated regulatory networks in fungal adaptation to DA-6-induced stress.

Collectively, these findings provide insights into the inhibitory mechanism of DA-6 against *A. flavus* and AF biosynthesis and highlight its potential application as antifungal agent to prevent fungal and mycotoxin contamination in food and agricultural products.

## Data Availability

The raw RNA sequencing data generated in this study have been deposited in the NCBI Sequence Read Archive (SRA) under BioProject accession number PRJNA1334134. All other data supporting the findings are included within the article and its supplemental material.

## SUPPLEMENTAL MATERIAL

Supplemental figures aem.00970-26-s0001.pdf Fig. S1 to S3.

Supplemental tables aem.00970-26-s0002.xlsx Tables S1 to S5.

ASM does not own the copyrights to Supplemental Material that may be linked to, or accessed through, an article. The authors have granted ASM a non-exclusive, world-wide license to publish the Supplemental Material files. Please contact the corresponding author directly for reuse.
