# Supplementary material for: Plant growth regulator diethyl aminoethyl hexanoate suppresses Aspergillus flavus growth and aflatoxin biosynthesis through disruption of redox homeostasis
Source: Appl Environ Microbiol. 2026 Sep 14;92(10):e00970-26. doi: 10.1128/aem.00970-26 (PMC13688885; doi:10.1128/aem.00970-26)
Supplement: Supplementary file 1 [file aem.00970-26-s0001.pdf]

Fig. S1. Transcriptomic analysis and validation of RNA-seq data by qRT-PCR analysis of differentially expressed genes in *Aspergillus flavus* following DA-6 treatment.

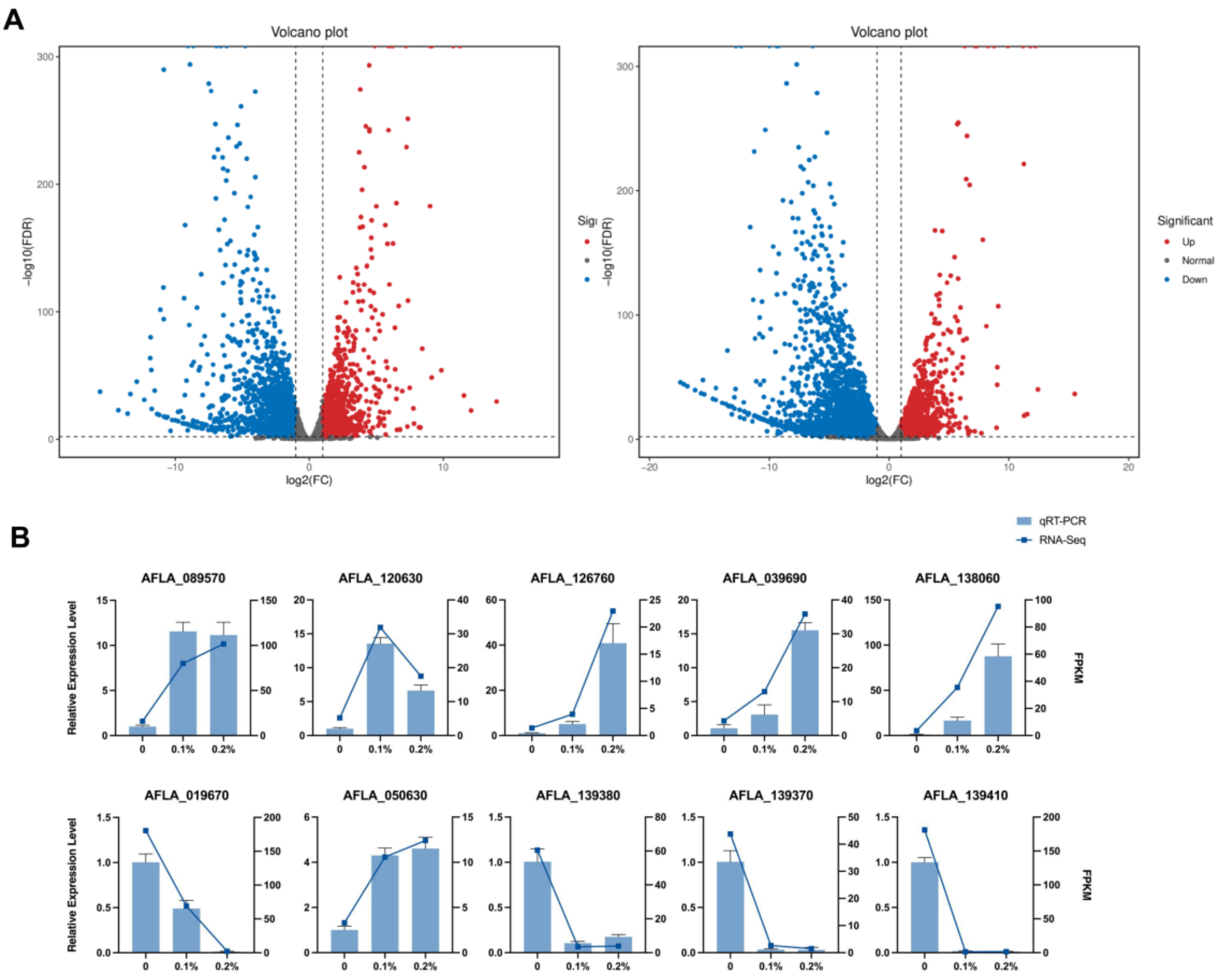

A. The DEGs in comparisons of 0.1% DA-6 (left) vs CK and 0.2% DA-6 vs CK (right) were showed in the volcano plots with red spots (up-regulation) and blue spots (down-regulation) and grey spots (no significantly changed genes). **B.** The expression trends of 10 differentially expressed genes determined by RNA-seq were confirmed through qRT-PCR. Bar chart illustrated the expression changes in response to DA-6 treatments for each selected gene according to measurements from RNA-seq and qRT-PCR. Data are shown as the mean ± SD (n=3).

Fig. S2. Functional enrichment analysis of differentially expressed genes (DEGs) in *Aspergillus flavus* treated with DA-6.

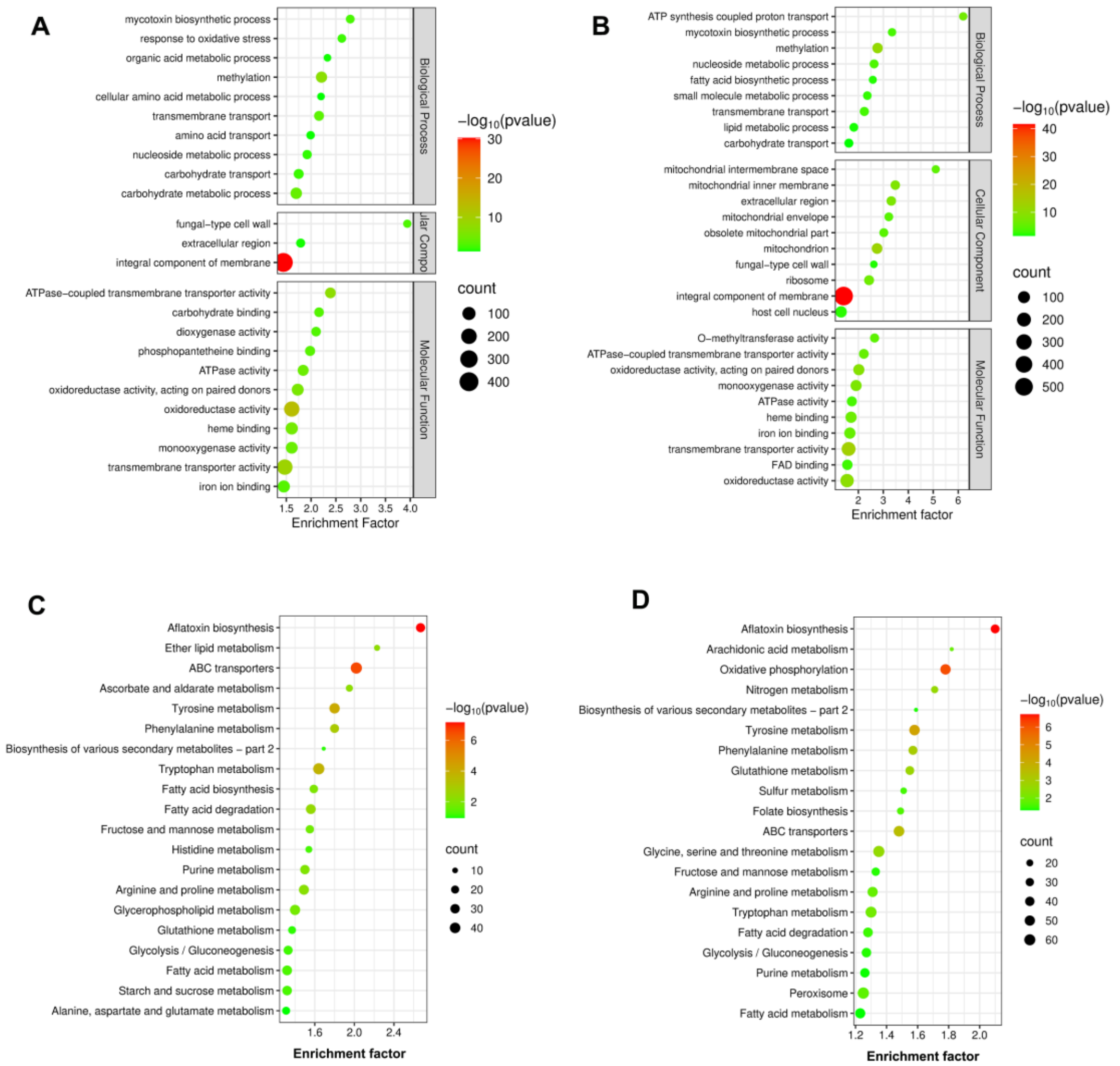

(A, B) Gene Ontology (GO) enrichment analysis of DEGs. The top 20 significantly enriched terms in the categories of biological process (BP), cellular component (CC), and molecular function (MF) are shown for (A) 0.1% and (B) 0.2% (v/v) DA-6 treatment, respectively.

(C, D) Kyoto Encyclopedia of Genes and Genomes (KEGG) pathway enrichment analysis of DEGs. The top 20 significantly enriched pathways for (C) 0.1% and (D) 0.2% (v/v) DA-6 treatment are shown. The enrichment factor and the -log10(*P*-value) represent the degree of enrichment and statistical significance, respectively.

Fig. S3. Construction and verification of Δ059960 and Δ084720 strains.

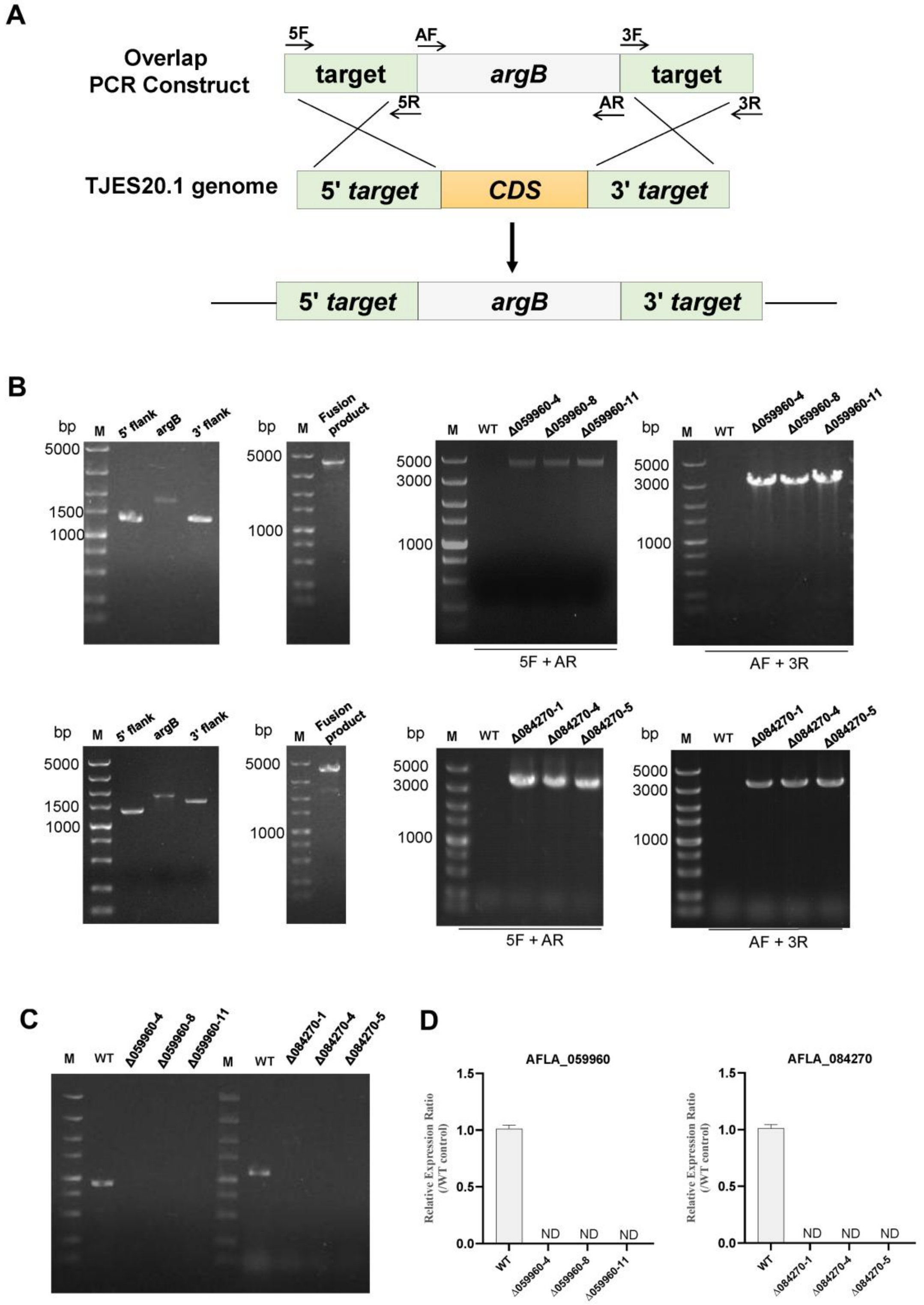

(A) Schematic diagram of the gene deletion strategy for the construction of Δ059960 and Δ084720 strains. **(B)** Diagnostic PCR verification of the putative transformants. **(C)** RT-PCR analysis of AFLA_059960 and AFLA_084720 gene expression in each strain from 24 h cultures were performed. **(D)** qPCR analysis for AFLA_059960 and AFLA_084720 gene expression in putative transformants. ND: not detected.
